# Evaluating the Effect of Parent–Child Interactive Groups in a School-Based Parent Training Program: Parenting Behavior, Parenting Stress and Sense of Competence

**DOI:** 10.1007/s10578-021-01276-6

**Published:** 2021-11-03

**Authors:** Sarah Buchanan-Pascall, Glenn A. Melvin, Michael S. Gordon, Kylie M. Gray

**Affiliations:** 1 Centre for Developmental Psychiatry & Psychology, Department of Psychiatry, School of Clinical Sciences at Monash Health, Monash University, Melbourne, Australia; 2 School of Psychology, Faculty of Health, Deakin University, Melbourne, Australia; 3 Centre for Educational Development, Appraisal and Research, University of Warwick, Coventry, UK; 4 Early in Life Mental Health Service, Monash Health, Melbourne, Australia

**Keywords:** Parent training, Parent behavior, Child behavior problems, Cluster-randomized trial

## Abstract

The Exploring Together program is a group-based parent training program that comprises separate parent, child, and teacher components, and a combined parent–child interactive component. A cluster-randomized trial design was used to compare the Exploring Together program with (Exploring Together; ET) and without (Exploring Together-Adapted; ET-Adapted) the parent–child interactive component. One hundred and thirty-six parents and their children (aged 5–10 years) with externalizing and/or internalizing problems participated in the trial, recruited from primary schools. There was a significant reduction in negative parenting behavior across both treatment groups (ET and ET-Adapted) but no significant improvement in positive parenting behaviors. Parenting self-efficacy improved significantly across both treatment groups however there was no significant change in parenting satisfaction or parenting stress. There was no consistent evidence of superiority of one version of the Exploring Together program over the other. Further investigation regarding treatment dosage and mastery of parenting skills associated with the program is warranted.

## Introduction

A substantial body of research has demonstrated the contribution of specific parenting behaviors and characteristics to the development and maintenance of child behavior problems [1, 2]. In particular, negative parenting behaviors comprise inconsistent discipline, harsh discipline, poor monitoring and supervision, have been repeatedly linked to child externalizing (e.g., aggression, oppositionality, defiance) problems [3] and show evidence of continuity across generations [4, 5]. Negative parenting behaviors provide a negative model of behavior, fail to promote pro-social child behavior, and impede development of adaptive social-cognitive skills [6]. Such deficits place children at risk of developing externalizing disorders during adolescence [7] and highlight the importance of identification and intervention to alter this developmental trajectory [8]. The association between negative parenting behaviors and child externalizing problems is well established [4, 9]. For instance, poor parental supervision and lack of involvement have been identified as significant risk factors for child conduct problems [10, 11]. Extreme discipline practices, including parental verbal aggression [12] and physical abuse [13] are also associated with child conduct problems. Even if the parent–child relationship is intermittently warm, punitive and physically harsh parenting behaviors are risk factors for the development of externalizing problems [14]. Studies focusing on bidirectional parent–child exchanges [15, 16] have found that children who exhibit more externalizing problems tend to have parents who exhibit higher rates of negative parenting behavior over time, and vice versa. Given the fundamental role that parenting plays in shaping child behavior, the need for early parenting interventions that reduce negative parenting behaviors and strengthen the parent–child relationship is highlighted.

Although negative or dysfunctional parenting behaviors have been consistently related to child externalizing problems, there is also research to suggest that negative parenting behaviors are associated with child internalizing problems [17, 18]. In a longitudinal, population-based survey completed by primary caregivers [19], negative parenting behavior was found to be one of the consistent and cumulative predictors of early childhood internalizing problems. Caron et al. [17] found that negative parenting behavior (e.g., threats, guilt induction) were associated with both child externalizing and internalizing problems, particularly for children whose parents also exhibited low warmth. Other researchers found that higher rates of negative parenting behaviors and lower rates of positive parenting behaviors were associated with more depressive symptoms in children [18]. This suggests the need for parenting interventions aimed at decreasing negative parenting behaviors.

Positive parenting behaviors (e.g., warmth, appropriate discipline, parental involvement) provide the foundations for healthy child development [20] and are associated with fewer child behavior problems [21]. Positive parenting behaviors emphasize the importance of promoting prosocial behaviors, such as self-regulatory skills, and minimizing psychologically harmful environments [22]. For example, children who grow up in environments characterized by warm, supportive and involved parents are less likely to develop antisocial and externalizing behavior problems even when faced with neighborhood deprivation, such as poverty and low socio-economic status [23]. Increased parental warmth, involvement, and nurturing behaviors are negatively associated with child internalizing problems such as anxiety [24, 25]. Positive parenting behaviors have been found to buffer children from the detrimental influences of harsh physical discipline [26]. Promoting positive parenting behaviors are a useful strategy in improving the welfare and psychosocial development of children [27, 28].

The influence of parenting stress on parenting behaviors and child behavior outcomes has also been a focus within the field of child development. Research suggests that parenting stress effects parenting behavior and the quality of dyadic parent–child interactions [29–31]. Further, the relationship between parenting stress and child behavior problems is bidirectional. That is, child externalizing and internalizing problems lead to increases in parenting stress over time, and high parenting stress leads to increases in externalizing and internalizing problems in children [32, 33]. Accordingly, parenting stress has been found to be effectively reduced by interventions that teach parents skills and strategies to effectively deal with their child’s behavior and that focus on the parent–child relationship [34].

Parenting sense of competence is another parenting construct that has been implicated in the relationship between parenting behaviors and child behavior outcomes [35]. Parenting sense of competence can be separated into two factors, parenting self-efficacy and parenting satisfaction. Parenting self-efficacy has been defined as the belief that parents hold about their ability to parent successfully [36], while parenting satisfaction refers to the degree to which parents feel frustrated or fulfilled in their parenting roles [37]. High maternal self-efficacy and parenting satisfaction have both been associated with positive parenting behaviors [38–40], which in turn, may lead to decreased child externalizing and internalizing problems [41]. Conversely, low parenting self-efficacy and parenting satisfaction have both been linked to negative parenting behaviors [42, 43], which are in turn correlated with child externalizing and internalizing problems [44]. Taken together, these findings demonstrate the value of parenting interventions that aim to enhance parenting sense of competence by teaching parents the skills they need to manage specific behavior problems.

Broadly, parent training interventions are focused, time-limited programs aimed at helping parents develop the parenting skills necessary to manage their child’s behavior and development. Many of these programs are informed by social learning theory and are based on the assumption that improvements in parenting behavior will lead to decreases in child problem behavior. Reviews have demonstrated that group-based parent training programs are among the most effective interventions for reducing child behavior problems [45–47]. Benefits of participating in a group with other parents can include gaining support and acceptance from other parents, and normalization of parent’s experiences [48]. Moreover, group-based parenting programs have been shown to improve parenting behavior and parenting self-efficacy as well as reduce parenting stress at least in the short-term [47, 49, 50].

Group-based parent training programs vary widely in regard to program content and delivery components [51]. However, programs generally use a range of strategies, including discussion, videotaped demonstrations, activities, and modelling of parenting behaviors and are typically delivered in 1–2 h weekly sessions over a period of 4 to 12 weeks [52]. Some group-based parent training programs include a parent–child interactive component, during which parents practice discipline skills and relationship enhancement skills with their child during treatment [53]. The inclusion of a parent–child interactive component is supported by studies demonstrating that changes in child behavior is activated by assisting parents to alter their own behavior and teaching parents how to interact more positively with their children via direct in vivo coaching strategies [54, 55]. Further, there is research evidence to support the use of parent training programs that involve in-session modelling, feedback and practice of new skills with parent’s own child [56–58]. There is limited evidence however concerning the additional benefits of incorporating a *parent–child interactive component* in group-based parenting programs with respect to parenting behavior, parenting stress and parenting sense of competence.

There are two parent training programs that involve an in vivo parent–child interactive component in treatment. The Exploring Together program is one example of a group-based parent training program that includes a parent–child interactive component [53, 59]. The Exploring Together program was developed to treat children at risk of developing internalizing and/or externalizing disorders, their parents and teachers. The program aims to develop parents' understanding of factors underlying their child's internalizing and/or externalizing problems, teach parents behavior management principles and techniques and assist parents to identify and regulate their own feelings. The Exploring Together program includes a parent–child interactive component in which parent–child relationship and interaction issues can be addressed as they arise, positive parent–child interaction can be modeled and encouraged, and problem solving and conflict resolution skills can be taught and practiced [53, 59]. The program treats parents and children as dyads and involves live coaching of parenting behaviors with both parent and child together in a group environment. The Exploring Together program has been found to significantly reduce negative parenting behaviors (e.g., authoritarian discipline, physical punishment) [59], as well as significantly improve parenting satisfaction [60]. Parent–Child Interactive Therapy (PCIT) is an individual parent training program for young children with externalizing and internalizing disorders that uses in vivo coaching of parental behaviors whilst the parent and child are together in treatment [54]. More recently, group-based adaptations of PCIT have been found to result in significantly reduced negative parenting behavior and parenting stress [61] and has shown promising evidence for reduction of child externalizing and internalizing problems [62–64].

While prior research supports the feasibility of using the live or video-feedback [65] coaching, it remains unclear whether the addition of a parent–child interactive coaching component improves outcomes within parent training programs relative to programs without an interactive component. Prior studies have identified positive parenting, negative parenting and behavior modification skills as the agents of change in reducing child behavior problems within parent training interventions [66, 67]. However, unlike other parenting-focused interventions, programs that incorporate a parent–child interactive component use in vivo coaching or video feedback to allow for an individualised approach to changing the dysfunctional parent–child relationship [56, 68]. Given that the inclusion of a parent–child interactive components requires greater resource allocation (e.g., additional staffing) further investigation into the added value of including such a component in treatment programs is warranted.

Research has shown that poor parenting quality is an important environmental factor that influences a young child’s behavior; it has almost twice the negative effect on child developmental outcomes of other known risks such as an impoverished environment [69]. Parent training programs aim to increase parental insight into the role of their own behaviors and responses to their child. The underlying assumption of parent training programs is that improving parenting behavior is the key mechanism of change in child behavior problems [70, 71]. In order to reduce problem behaviors and enhance the development and wellbeing of children, it is therefore essential that parent training programs successfully change parenting behavior. Prior work from the same research project found that the Exploring Together program [72] significantly reduced child externalizing and internalizing problems, both with and without the parent–child interactive component. Given that parenting behaviors are the assumed mechanism of change in child behavior outcomes, it is necessary to evaluate outcomes in terms of this construct.

The current study aimed to compare the effectiveness of two versions of the Exploring Together program for improving parenting behavior, parenting stress and parenting sense of competence, associated with (Exploring Together; ET) and without (Exploring Together-Adapted; ET-Adapted) the parent–child interactive component. The study also aimed to compare parenting satisfaction with the two versions of the program at post intervention. It was hypothesized that (1) there would be a reduction in negative parenting behaviors and improvement in positive parenting behaviors across treatment (ET, ET-Adapted) over time (baseline, post intervention, 6- and 12-month follow-up) and (2) there would be a reduction in parenting stress and improvement in parenting sense of competence across treatment (ET, ET-Adapted) over time (baseline, post intervention, 6- and 12-month follow-up). It was also hypothesised that (1) the reduction in negative parenting behaviors and improvement in positive parenting behaviors would be greater for parents in the ET program compared to the ET-Adapted program over time (baseline, post intervention, 6- and 12-month follow-up) and (2) the reduction in parenting stress and improvements in parenting sense of competence would be greater for parents in the ET program compared to the ET-Adapted program over time (baseline, post intervention, 6- and 12-month follow-up).

## Method

### Design

A cluster-randomized trial was conducted within the Child and Adolescent Mental Health Services and Schools Early Action (CASEA) program at Monash Health Early in Life Mental Health Service, Victoria, Australia [72]. School was the unit of randomization; participating primary schools were randomly allocated to treatment condition. This trial was designed in accordance with CONSORT guidelines [73] and registered with the Australian New Zealand Clinical Trials Registry (www.anzctr.org.au; Trial Number: ACTRN1261700152 3392). Approval was obtained from the Monash University and Monash Health Human Research Ethics Committees.

### Participants

**Fig. 1:**
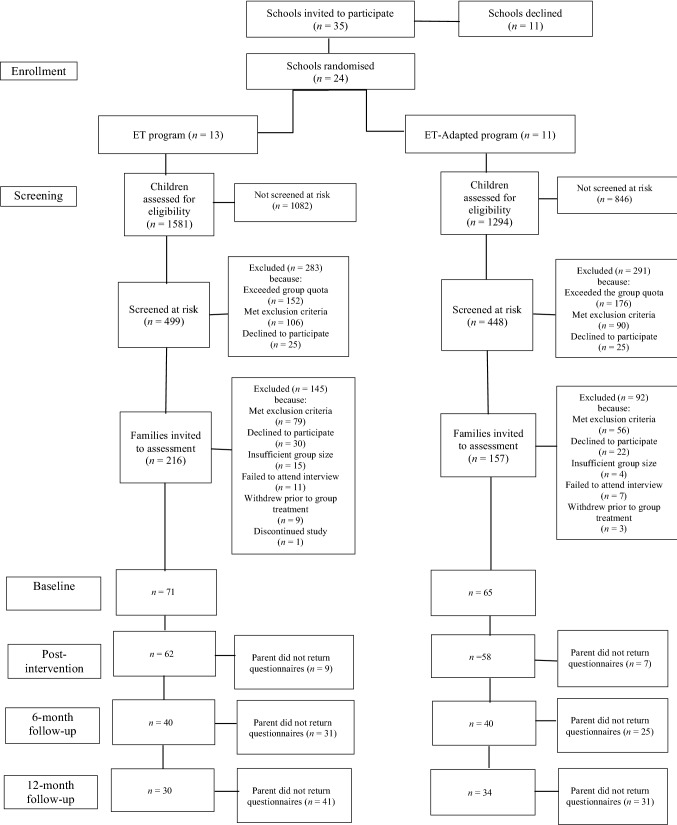
Participant flow

One hundred and forty-eight (*n* = 148) parent/child dyads were invited to participate in treatment. Twelve (*n* = 12) parent/child dyads withdrew prior to treatment, resulting in one hundred and thirty-six (ET *n* = 71; ET-Adapted *n* = 65) parents and their children aged 5 to 10 years who participated in the two treatment groups (see Fig. 1 for participant flow). Baseline (pre-treatment) characteristics of participants in the two treatment groups (ET, ET-Adapted) are presented in Table 1. There was a statistically significant difference between groups with respect to parent gender (Fisher’s exact probability test; *p* = 0.03) with no male parents in the ET-Adapted group. An independent-samples *t*-test indicated that parents in the ET group were significantly older than parents in the ET-Adapted group (ET: *M* = 38.83, *SD* = 6.33; ET-Adapted: *M* = 36.75, *SD* = 4.67; *t* (127.89) = -2.18, *p* = 0.03). Gender and age of parent participants was controlled for in the main analysis.

**Table 1:** Baseline characteristics of participants by treatment group

	ET *n* = 71	ET-adapted *n* = 65	*t* or χ^2^	*p*
Parent characteristics				
Sex				
Male	6 (8.5%)	0 (0.0%)	χ^2^ (1) = 3.92	0.03*
Female	65 (91.5%)	65(100.0%)		
Age (years) *M* (*SD)*	38.83(6.33)	36.75 (4.67)	*t* (127.89) = − 2.18	0.03*
Parent marital status				
Married/cohabitating	49 (69.0%)	51 (78.5%)	χ^2^ (2) = 2.95	*ns*
Separated/divorced	13 (18.3%)	11 (16.9%)		
Single	9 (12.7%)	3 (4.6%)		
Parent education				
Did not complete high school	18 (25.4%)	18 (28.6%)	χ^2^ (3) = 4.56	*ns*
Completed high school	12 (16.9%)	10 (15.9%)		
Tertiary qualification	25 (35.2%)	29 (46.0%)		
University degree	16 (22.5%)	6 (9.5%)		
Child characteristics				
Age (years)				
M (SD)	7.70 (1.18)	7.68 (1.07)	*t (134)* = *−* *0.21*	*ns*
Range	5.20–10.23	5.58–9.62		
Gender				
Male	47 (66.2%)	39 (60.0%)	*χ*^2^* (1)* = *0.33*	*ns*
Female	24 (33.8%)	26 (40.0%)		
Family characteristics				
Description of household				
One parent	20 (21.5%)	14 (21.5%)	χ^2^ (1) = 0.48	*ns*
Two parent	51 (71.8%)	51 (78.5%)		
Total family income (weekly)				
$0–$769	14 (19.7%)	14 (21.5%)	χ^2^ (3) = 3.25	*ns*
$770–$1729	26 (36.6%)	32 (49.2%)		
$1730–$2211 or more	20 (28.2%)	12 (18.5%)		
Don’t know or missing	11 (15.5%)	7 (10.8%)		

*p < 0.05

### Procedure

Primary schools were recruited from the south-east metropolitan suburbs of Melbourne and the Mornington Peninsula region, Victoria, Australia. Informed written consent was obtained from all schools and parents of children who participated in the study. Monash Health Child and Adolescent Mental Health Services and Schools Early Action (CASEA) program clinicians invited 35 primary schools to participate in the trial, of which 24 (69%) schools (government *n* = 20; Catholic *n* = 4) consented to participate. Once schools consented to participate they were randomly allocated to type of treatment program (ET or ET-Adapted). An independent research officer used a computer-generated random allocation procedure to assign each school to either the ET or ET-Adapted program. Participants were not blind to school allocation. When randomisation of school to treatment condition was completed, the program was announced in the school newsletter. A letter was then provided to parents of children in Preparatory year to Grade Three, explaining the program in more detail and inviting them to participate.

#### Participant Selection Criteria

Children with significant externalizing and/or internalizing problems were eligible for participation in the treatment trial. Parents of all children in the first four years of primary school were invited to complete and return the Parent version of the Strengths and Difficulties Questionnaire (SDQ) [74] in a sealed envelope, along with a signed consent form, to the child’s classroom teacher. The Teacher version of the SDQ was completed for children whose parents had consented to participate. Eligibility for the treatment program was determined on the basis of a two-step selection process. In the first step of the selection process, children were eligible for possible participation in the treatment program if they scored in the borderline—abnormal range on the Total Difficulties score (≥ 14 on Parent SDQ and/or ≥ 12 on Teacher SDQ), Externalizing score (combined Conduct Problems Score, Hyperactivity Score, & Peer Problems Score) (≥ 12 on Parent SDQ and/or ≥ 13 on Teacher SDQ), or Internalizing score (Emotional Symptoms Score) (≥ 4 on Parent SDQ and/or ≥ 5 on Teacher SDQ) on the Parent and/or Teacher SDQ. Monash Health CASEA program clinicians met with school leadership staff and classroom teachers to ascertain if any child eligible on the SDQ criteria met exclusion criteria. Children with a pre-existing diagnosis (e.g., Autism Spectrum Disorder, intellectual disability, or severe behavior disorder) and who qualified for government-funded specialist education support [75] were excluded. Children who were recognised to require urgent treatment for severe mental illness (e.g., non-suicidal self-injury, family crisis, child protective concerns) were excluded and referred to an appropriate service for treatment. Appropriate referrals (e.g., community mental health services) were provided to parents of children not accepted into the treatment program.

In the second step of the selection process, parents of eligible children were invited to an interview to determine parent commitment and readiness to participate in the treatment program. Parent–child dyads who were unable to attend any or substantial proportion of the treatment sessions, had recent or current significant change in family circumstances (i.e., new baby, parental divorce, death in family), parental mental health problems, an intellectual disability or insufficient English for group participation, were currently engaged in another group parenting program, or, were currently involved in child protection case management were excluded. The remaining short-listed eligible children were then discussed in clinical review by Monash Health CASEA program clinicians using all clinical information gathered (i.e., SDQ results, teacher interview, family assessment). Selection of parent/child dyads was based on severity of the child’s behavior problems, consideration of potential group cohesion (i.e., sufficient mix of social, externalizing and internalizing problems, gender mix, age mix), and willingness to engage in the program. Between five and eight consenting parent–child dyads were invited to participate in the treatment program.

A total of 947 children met the SDQ eligibility criteria; 148 of these children and their parents were invited to participate in treatment (refer to Fig. 1 for participant flow). Children who were invited to participate in treatment (*n* = 148) were compared with children who met the SDQ eligibility criteria but did not receive treatment (i.e., due to group capacity of maximum eight parent/child dyads, declining or discontinuing; *n* = 799). There were no statistically significant differences between children who were invited to participate in treatment compared with children who met SDQ criteria but were not invited to receive treatment with regard to child gender, parent-reported SDQ Internalizing scores or teacher-reported SDQ Internalizing scores (all *p* > 0.05). However, children who were invited to participate in treatment were significantly older [*t* (830) = 3.66, *p* = 0.0003] compared to children who met SDQ criteria but were not invited to receive treatment. Also, children who were invited to participate in treatment were from families that had significantly higher Index of Relative Socio-economic Disadvantage [76] scores [*t* (945) = 4.80, *p* < 0.0001] compared to children who met SDQ criteria but were not invited to receive treatment. This suggests that children who were invited to participate in treatment were less socio-economically disadvantaged compared to children who met SDQ criteria but were not invited to receive treatment. Children who were invited to participate in treatment had significantly higher teacher-reported SDQ Externalizing scores [*t* (847) = 2.54, *p* = 0.01] and significantly higher parent-reported SDQ Externalizing scores [*t* (804) = 4.52, *p* < 0.0001] compared with children who met SDQ criteria but were not invited to receive treatment. Children who were invited to participate in treatment had significantly higher teacher-reported SDQ Total Difficulties scores [*t* (836) = 2.66, *p* =  < 0.05] and significantly higher parent-reported SDQ Total Difficulties scores [*t* (791) = 3.95, *p* < 0.05] compared with children who met SDQ criteria but were not invited to receive treatment.

Of the 136 parent/child dyads who participated in treatment, 14% (*n* = 19) of children met the inclusion criteria for only internalizing problems, 8% (*n* = 11) of children met the exclusion criteria for only externalizing problems, and 78% (*n* = 106) of children met the inclusion criteria for both externalizing and internalizing problems.

### Treatment Program

The Exploring Together program [77] is a short-term, group-based parent training for children with externalizing and internalizing problems. The Exploring Together program is a treatment program that involves children, their parent, and teachers [53]. The program is used in the early primary school aged period, as this is the period of development when externalizing and internalizing problems are becoming of significant concern to parents and teachers [78]. The theoretical underpinning of the Exploring Together program draws upon a number of psychological theories, including social learning [79], cognitive-behavioral [80] and attachment theories [81]. The program focuses on reducing child externalizing and internalizing problems, improving parenting behaviors and strengthening parent–child and teacher–child interactions. The original manualised program [77] comprises three treatment group components, namely parent–child interactive groups, child groups, and parent groups, as well as two meetings for partners or support persons and two meetings for teachers throughout the program.

Based on social learning principles [82], Exploring Together aims to teach parents to increase positive interactions with their children and to reduce the use of coercive and inconsistent parenting practices. This helps the parent and child to form an important connection from which a positive relationship can develop, and from which discipline practices are acceptable and meaningful for both parties. The cognitive-behavioral approach to parenting is represented in the Exploring Together program with its emphasis on exploration of antecedents of children’s behavior and of the consequences of parental action or inaction, rewards and punishments, as well as formulation and application of behavior management plans [83].

Children with behavior problems often have a history of difficult parent–child relationships that contribute to and maintain their behavior problems [84]. Guided by attachment theory [81] and taking into account the attachment difficulties that often underlie behavior problems, the Exploring Together program aims to help parents and children to develop positive, safe interactions with each other. During the parent–child interactive component, parents practice in vivo relationship enhancement skills with their child during treatment [53]. The process is designed to improve communication and understanding between parent and child. The ability of parents to sit with and contain children during a frightening and stressful period (e.g., when feeling out of control and overwhelmed by their emotions) helps the child to feel safe and supported by their parent.

Two versions of the Exploring Together early primary school program [77] were implemented (ET and ET-Adapted) in the current study. The two treatments (ET, ET-Adapted) differed in that the ET program included parent–child interactive groups whereas the ET-Adapted program omitted this component. The parent–child interactive groups included in the ET program involved direct parent–child (dyad) work to address relationship and parent–child interaction issues as they arose, teach and practice problem solving and conflict resolution skills, and encourage a cooperative parent–child relationship. Additionally, leaders modelled positive and appropriate interactions and parent–child dyads were coached through behavior management issues.

Treatment structure and treatment session times for each version of the program (ET, ET-Adapted) are presented in Table 2. Both versions of the program consisted of 9 (weekly) sessions to coincide with school terms and were provided free to participants. During the study, one parent was required to be able to attend the 9-week group program. Groups were conducted during school hours at the participating primary school. Each version of the program consisted of all of the content covered in the original manualized version of the Exploring Together early primary school program [77], including completion of weekly Mystery Mission (homework) tasks. In the current study, parents were taught Emotion coaching principles [85] throughout both versions of the program. Emotion coaching principles were not taught in the original manualized version of the Exploring Together program [77]. Emotion coaching involves parents talking with children about their feelings, and offering children strategies for coping with emotionally difficult situations. Research has shown that children who are emotionally coached have fewer internalizing and externalizing problems, including problems with anger, anxiety, and disruptive behavior [85–87].

**Table 2:** Weekly session structure and treatment session duration for the exploring together program

	ET program	ET-Adapted program
Treatment time	9 sessions, 2 h per session Total: 18 h	9 sessions, 1.5 h per session Total: 13.5 h
Weekly (9 week) session structure	Sessions 1–9 First parent–child interactive group (40 min, 4 leaders) Child group (1 h, 2 leaders) Parent group (1 h, 2 leaders) Second parent–child interactive group (20 min, 4 leaders)	Sessions 1–9 Child group (1.5 h, 2 leaders) Parent group (1.5 h, 2 leaders)

During both programs (ET and ET-Adapted), parenting topics covered in the parent’s group included: understanding child behavior in context of normal developmental stages; ABC (Antecedents, Behavior, Consequences) model of behavior management; behavior management plans; natural and logical consequences; rules and limit setting; managing strong emotions; special time and self-care; skill generalisation and relapse prevention; family of origin; and, assertiveness/self-esteem.

For both programs (ET and ET-Adapted), the aim of the children’s group was to reduce children's aggressive and/or withdrawn behaviors whilst improving peer interactions. The children’s group focused on teaching the children anger management, pro-social skills, perspective taking, conversation skills, problem-solving skills, feelings recognition, assertion skills, decision-making, and social perception. This was done through group activities such as games, stories and role-play.

During the ET program, there were two parent–child interactive group sessions each week (see weekly program structure in Table 2). Each week, the first interactive group was 40-min in length and enabled children to settle into the program in the presence of their parents. It provided an opportunity for parents to model appropriate group behaviors for children and leaders to model behaviors for parents. It also provided parents with an opportunity to work with their child on issues arising from the past week. Following the first interactive group, parents and children then separated to attend concurrent parent and child groups before reuniting for the second interactive group. Each week, the second interactive group was 20-min in length and allowed parents and children to handle reunion after separation, as well as providing an opportunity for sharing and other behavior problems to be addressed within the group context. At the end of the second interactive group session, Weekly Mystery Mission (homework) tasks were explained and distributed to parent–child dyads.

During both versions of the program, each participating child’s teacher was invited to two group meetings, led by project clinicians. The meetings aimed to promote a consistent approach in the management of the child across the different systems in the child's life. The meetings also provided the opportunity for two-way feedback between teachers and group leaders. Two parent evenings were held throughout both versions of the program. The group-based meetings for parents were led by project clinicians and attended by both parents or by the main attending parent and a support person. The meetings aimed to elicit the partners’ view of the child and family and the problems leading to inclusion in the group. The meetings also intended to encourage adults to work together and support each other in disciplining and nurturing their child.

Following treatment, parent participants from both programs (ET and ET-Adapted) were invited to attend a group booster session that coincided with the 6-month follow-up assessment. The purpose of the booster session was to assess progress, reinforce parenting strategies, and provide an opportunity to troubleshoot any problems that may have arisen. Parents either completed the outcome measures during the booster session or via post. Thirteen (*n* = 13, 9.6%) parents were not offered the opportunity to attend a booster session due to staffing resources.

#### Treatment Fidelity

The treatment groups were led by Monash Health CASEA program clinicians. Treatment fidelity was enhanced by all group leaders attending a 2-day training workshop with the Exploring Together program authors or training through co-leading a group with a previously trained Monash Health CASEA program clinician. Treatment manuals for both programs (ET, ET-Adapted) included outlines of the core therapeutic content to be addressed in each treatment session. To assess therapist fidelity to core therapeutic content, fidelity checklists of a random subsample (16%) of treatment sessions were anonymously completed by Monash Health CASEA program clinicians.

### Measures

Treatment efficacy was assessed via parent self-report of parenting behavior, parenting stress and parenting sense of competence. Outcome measures were collected at baseline (Time 1), post intervention (Time 2), 6- (Time 3) and 12-month (Time 4) follow-up. Parents completed a demographic questionnaire at baseline (Time 1) and a cognitive screen (Kaufman Brief Intelligence Test, Second Edition; KBIT-2) [85] was completed with each child at baseline (Time 1). The Index of Relative Socio-economic Disadvantage [73] was used to measure school socio-economic disadvantage at baseline (Time 1). A parent satisfaction questionnaire was completed at post intervention (Time 2) only.

#### Demographic Information

A demographic questionnaire was completed by parents at baseline (Time 1) in order to gather information (e.g., age and gender of family members, parent education, occupation, and income levels) about the participants and their families.

#### Index of Relative Socio-economic Disadvantage (IRSD)

Socio-Economic Indices for Areas (SEIFA) [76] ranks areas in Australia according to relative socio-economic advantage and disadvantage. One of these indices, the Index of Relative Socio-economic Disadvantage, ranks areas on a continuum from most disadvantaged to least disadvantaged. An Index of Relative Socio-economic Disadvantage score was determined for each participant in this study according to the geographic area where the child’s participating school was located. A low score on this index indicates a high proportion of relatively disadvantaged people in an area.

#### Kaufman Brief Intelligence Test-2 (KBIT-2)

The KBIT-2 is a brief, screening measure for verbal and nonverbal intelligence in individuals aged 4 to 90 years and has sound psychometric properties [88, 89]. Children (*n* = 5) were excluded from this study if their KBIT-2 Overall (or Composite) IQ standard score was ≤ 70 (i.e. more than 2*SD* below mean).

#### Parent Satisfaction with the Program

Parents completed a parent satisfaction questionnaire at post intervention to measure how the program was received. Parents rated how useful they found or how satisfied they were with various aspects of the program on a 5-point Likert scale ranging from 1 (i.e., *not at all*) to 5 (i.e., *very*). The mean item rating for parents was used to assess treatment satisfaction. Example items included *What was your level of satisfaction with the children’s group?* and *Were the activities in this group helpful in improving your relationship with your child?* Internal consistency for the parent satisfaction questionnaire was measured at Time 2 (α = 0.94).

#### Parenting Behavior: Alabama Parenting Questionnaire (APQ)

The APQ [90] is a 42-item scale that measures parenting behavior across five different parenting domains utilising a 5-point scale: never, almost never, sometimes, often, and always. Parenting behaviors as measured by the APQ are associated with child internalizing and externalizing problems [91]. Past research has indicated that the five parenting domains of the APQ can be combined into two composites scores (i.e., positive and negative parenting) [90, 92]. In this study, positive parenting behaviors were measured using the involvement and positive parenting subscales on the APQ, while negative parenting behaviors were measured using the poor monitoring/supervision, inconsistent discipline, and corporal punishment subscales [90, 93]. Adequate test–retest reliability, internal consistency (subscales ranged from α = 0.46 to 0.80) and convergent validity have been reported [90]. Internal consistency for APQ positive parenting was measured at Times 1 (α = 0.79), 2 (α = 0.84), 3 (α = 0.85), and 4 (α = 0.84). Internal consistency for APQ negative parenting was measured at Times 1 (α = 0.76), 2 (α = 0.76), 3 (α = 0.79), and 4 (α = 0.76).

#### Parenting Stress: Parenting Stress Index-Short Form (3rd ed.) (PSI-SF)

The PSI-SF [94] is a 36-item parent self-report questionnaire designed to identify potentially dysfunctional parent–child systems. The PSI-SF items measure parental distress (PD-SF), parent–child dysfunctional interaction (PCDI-SF) and difficult child behavior (DC-SF). The Total Score of the PSI-SF reflects the stresses reported in the areas of personal parental distress, stresses derived from the parent’s interaction with the child, and stresses that result from the child’s behavioral characteristics. In the current study, the PSI-SF total score was used to measure change in overall level of parenting stress experienced by an individual. Internal consistency for PSI-SF total score was measured at Times 1 (α = 0.91), 2 (α = 0.93), 3 (α = 0.96), and 4 (α = 0.96).

#### Parenting Sense of Competence: Parenting Sense of Competence Scale (PSOC)

The PSOC [37] is a 16-item self-report questionnaire designed to measure parent’s satisfaction and efficacy in the parenting role. The PSOC consists of 17 items answered on a 6-point scale ranging from “strongly disagree” to “strongly agree”, with nine items under Satisfaction and seven items under Efficiency. In this study, parenting sense of competence was measured using the parenting efficacy and parenting satisfaction subscale scores on the PSOC. Internal consistency for PSOC parenting efficacy was measured at Times 1 (α = 0.73), 2 (α = 0.73), 3 (α = 0.74), and 4 (α = 0.85). Internal consistency for PSOC parenting satisfaction was measured at Times 1 (α = 0.74), 2 (α = 0.77), 3 (α = 0.78), and 4 (α = 0.82).

### Analysis

Multilevel model analyses were conducted to assess the impact of treatment condition (ET, ET-Adapted) from baseline to 12-month follow-up on outcome variables, taking into account the random effect of school. This followed from the nested data structure (on average, seven children nested within schools) and the presence of moderate intraclass correlation coefficients (ranging from 0.16 to 0.55 for the APQ, 0.07 to 0.41 for the PSOC, and 0.72 to 0.80 for the PSI-SF). The main results were calculated using available data from all participants who entered the study (i.e., irrespective of number of sessions completed). Raw scores were used in all analyses. The main analysis was conducted using Mixed Linear Models (MLM) with SPSS 23. A MLM was fit with intercept and school as random effects and repeated effect of time for children nested in schools. A random effect (intercept) of school was included to account for school-to-school differences that induce correlation among scores for students within a school. Step 1 of the model building involved construction of a baseline random intercept model for each outcome measure. Best model fit for the null model was determined by the smallest Akaike Information Criterion (AIC) index, and achieved using unstructured covariance structure and maximum likelihood estimation [95]. At step 2, key variables (i.e., treatment and time) were added to the model as fixed effects. All analyses controlled for school, child gender, child age, IQ composite score (K-BIT-2), parent gender, parent age, and family income. Alpha was set to *p* < 0.05 for all analyses. Separate effect sizes of changes that occurred within ET and ET-Adapted (relative to each other) were calculated [96]. As a guide, effect sizes (*d*) values can generally be interpreted as follows: 0.01 to 0.2 (very small to small), 0.2 to 0.5 (small to medium), and 0.5 to 0.8 (medium to large) [97].

## Results

### Attrition Analysis

A number of parents failed to return measures at follow-up time points (see Fig. 1). However, there were no statistically significant differences between groups on baseline mean scores on all outcome variables for parents who did and did not complete assessment measures at post intervention, 6- and 12-month follow-up, suggesting no bias in missing follow-up data. Prior work from the same research project [72] found no statistically significant differences between groups on baseline mean scores of parent-reported child externalizing or internalizing problems for parents who did and did not complete assessment measures at post intervention, 6- and 12-month follow-up, suggesting no bias in missing follow-up data.

### Treatment Attendance

On average program attendance by parents and children was high, with more than 80% of parents and children attending seven or more (≥ 78%) weekly treatment sessions across both programs (ET, ET-Adapted) and no statistically significant difference between treatment groups on any measure of program attendance.

There was no statistically significant difference between treatment groups (ET, ET-Adapted) for parents who were offered versus parents who were not offered the opportunity to attend a booster session at the 6-month follow-up, χ^2^ (1, *N* = 136) = 1.09, *p* = 0.30. Of those parents offered a booster session at 6-month follow-up, there was no statistically significant difference between treatment groups (ET, ET-Adapted) for attendance at the booster session, χ^2^ (2, *N* = 136) = 1.66, *p* = 0.44. Parents who were and were not offered a booster session at the 6-month follow-up did not differ significantly on 12-month follow-up scores on any of the outcome measures (APQ positive parenting: *U* = 277, *z* = 0.67, *p* = 0.50; APQ negative parenting: *U* = 220.50, *z* = − 0.36, *p* = 0.72; PSOC efficacy: *U* = 176.50, *z* = − 0.61, *p* = 0.54; PSOC satisfaction: *U* = 180.50, *z* = − 0.52, *p* = 0.60; PSI-SF total: *U* = 212.50, *z* = 0.23, *p* = 0.82). Results suggest no bias in the 12-month follow-up data based on whether parents were offered the opportunity to attend a booster session at the 6-month follow-up assessment.

### Treatment Fidelity

Thirty (16%) treatment sessions (ET program = 18; ET-Adapted program = 12) were reviewed for treatment fidelity. There was no significant difference between treatment groups in overall clinician adherence to core therapeutic content for the parent group sessions (ET program = 86%; ET-Adapted program = 89%) or child group sessions (ET program = 88%; ET-Adapted program = 93%). Overall clinician adherence to core therapeutic content was 77% for the parent–child interactive groups encompassed within the ET program.

### Treatment Effects on Parenting Behavior, Parenting Stress and Sense of Competence

A summary of the adjusted means and standard errors for each outcome variable at baseline, post intervention, 6- and 12-month follow-ups are presented in Table 3.

**Table 3:** Multi-level mixed effects modeling: adjusted^a^ means and standard errors of outcome variables

	Baseline		Post intervention		6-month follow-up		12-month follow-up	
	ET	ET-adapted	ET	ET-adapted	ET	ET-adapted	ET	ET-adapted
	*M (SE)* *n*		*M (SE)* *n*		*M (SE)* *n*		*M (SE)* *N*	
APQ positive	65.48 (0.86) 71	66.03 (0.90) 64	65.84 (0.98) 62	65.95 (1.02) 58	66.00 (1.01) 42	66.36 (1.04) 40	65.83 (1.03) 29	67.29 (1.01) 34
APQ negative	33.70 (0.92) 71	33.59 (0.96) 65	30.91 (0.80) 61	30.79 (0.83) 57	31.12 (0.94) 40	30.23 (0.96) 40	30.40 (0.88) 28	29.29 (0.86) 34
PSOC satisfaction	36.69 (0.94) 71	36.85 (0.99) 64	37.08 (0.92) 62	39.09 (0.96) 58	37.25 (1.04) 40	39.42 (1.06) 40	37.14 (1.13) 27	41.38** (1.10) 32
PSOC efficacy	32.71 (0.88) 70	34.20 (0.91) 64	35.68 (0.82) 60	35.17 (0.85) 57	34.58 (0.91) 40	35.74 (0.94) 39	34.63 (1.24) 27	37.23 (1.17) 32
PSI-SF total	81.90 (2.39) 62	80.00 (2.44) 59	79.89 (2.52) 50	76.12 (2.55) 51	80.32 (3.41) 35	74.50 (3.40) 37	76.83 (3.46) 23	71.76 (3.30) 31

All analyses used raw scores

*M* mean; *SE* standard error; *APQ* Alabama Parenting Questionnaire; *PSI-SF* Parent Stress Index-Short Form; *PSOC* Parenting Sense of Competence; *ET* Exploring Together; *ET-Adapted* Exploring Together-Adapted

^a^All scores adjusted for school, child gender, child age, IQ composite score (K-BIT-2), parent gender, parent age, and family income

**Significantly different from adjusted* mean of ET at 12-month follow-up, *p* < 0.01

#### Change Over Time

Model estimates, including significance figures for the main effect of time are presented in Table 4. A significant main effect of time indicated parents in both treatment groups showed improvements in parent reported negative parenting behavior at post intervention (*β* = − 2.79, *t* (109.93) = − 4.08, *p* < 0.001), 6-month follow-up (*β* = − 2.58, *t* (92.78) = − 3.61, *p* < 0.001), and 12-month follow-up (*β* = − 3.30, *t* (67.79) = − 4.33, *p* < 0.001). A significant main effect of time indicated parents in both treatment groups showed improvements in parent reported self-efficacy at post intervention (*β* = 2.97, *t* (108.42) = 4.65, *p* < 0.001) and 6-month follow-up (*β* = 1.87, *t* (84.39) = 2.53, *p* = 0.01). There was a non-significant main effect of time on all other outcome variables.

**Table 4:** Multi-level mixed effects modeling: main effect of time for outcome variables

Measure	Time	Main effect of time					
		*Β*	SE	95% CI	*Df*	*t*	*p*
APQ positive	Post intervention	0.37	0.88	− 1.38, 2.11	106.90	0.42	0.68
	6-month intervention	0.52	0.84	− 1.15, 2.19	72.19	0.62	0.53
	12-month intervention	0.35	0.87	− 1.40, 2.10	60.80	0.40	0.69
APQ negative	Post intervention	− 2.79	0.68	− 4.14, − 1.43	109.93	− 4.08	< 0.001***
	6-month intervention	− 2.58	0.72	− 4.00, − 1.16	92.78	− 3.61	< 0.001***
	12-month intervention	− 3.30	0.76	− 4.82, − 1.78	67.79	− 4.33	< 0.001***
PSOC satisfaction	Post intervention	0.39	0.82	− 1.23, 2.01	106.87	0.48	0.63
	6-month intervention	0.56	0.87	− 1.16, 2.28	89.64	0.65	0.52
	12-month intervention	0.44	0.99	− 1.52, 2.41	72.24	0.45	0.66
PSOC efficacy	Post intervention	2.97	0.64	1.71, 4.24	108.42	4.65	< 0.001***
	6-month intervention	1.87	0.74	0.40, 3.34	84.39	2.53	0.01*
	12-month intervention	1.92	1.17	− 0.42, 4.26	60.55	1.64	0.11
PSI-SF total	Post intervention	− 2.01	1.78	− 5.54, 1.52	87.30	− 1.13	0.26
	6-month intervention	− 1.58	2.73	− 7.02, 3.86	65.60	− 0.58	0.57
	12-month intervention	− 5.07	2.55	− 10.19, 0.05	54.46	− 1.98	0.052

All analyses used raw scores

All scores adjusted for school, child gender, child age, IQ composite score (K-BIT-2), parent gender, parent age, and family income

*Β* beta coefficient, the degree of change in the outcome variable for every 1-unit of change in the predictor variable. If the beta coefficient is positive, the interpretation is that for every 1-unit increase in the predictor variable, the outcome variable will increase by the beta coefficient value. If the beta coefficient is negative, the interpretation is that for every 1-unit increase in the predictor variable, the outcome variable will decrease by the beta coefficient value

*APQ* Alabama Parenting Questionnaire; *ET* Exploring Together; *ET-Adapted* Exploring Together-Adapted; *PSI-SF* Parent Stress Index-Short Form; *PSOC* Parenting Sense of Competence

*p < 0.05, **p < 0.01, ***p < 0.001

#### Group (Treatment) by Time Interactions

Model estimates, including significance figures for the interaction between group (treatment) and time, as well as effect sizes, are presented for all outcome variables in Table 5. There was a statistically significant group by time interaction for parent reported self-efficacy (*β* = − 2.01, *t* (107.74) = − 2.18, *p* = 0.03) at post intervention, with greater improvement in parent reported self-efficacy for parents in the ET group. There was a statistically significant group by time interaction for parent reported parenting satisfaction (*β* = 4.09, *t* (71.48) = 2.98, *p* = 0.004) at the 12-month follow-up, with greater improvement in parent reported parenting satisfaction for parents in the ET-Adapted group. Otherwise, group by time interactions were non-significant for all other outcome variables at all time points. Effect size values comparing the ET program to the ET-Adapted program across all outcome variables at all time points ranged from very small-to-medium (*d* = − 0.002 to *d* = 0.61) (see Table 5).

**Table 5:** Multi-level mixed effects modeling: test of interaction and effect size data for parent-reported outcome variables

Measure	Time	Condition	Test of interaction^a^						
			*Β*	*SE*	95% CI	*Df*	*t*	*p*	*d*
APQ positive	Post intervention	ET ET-Adapted	− 0.45	1.28	− 2.98, 2.08	108.05	− 0.36	0.72	− 0.07
	6-month intervention	ET ET-Adapted	− 0.20	1.20	− 2.60, 2.20	73.11	− 0.16	0.87	− 0.03
	12-month intervention	ET ET-Adapted	0.91	1.21	− 1.52, 3.34	58.31	0.75	0.46	0.14
APQ negative	Post intervention	ET ET-Adapted	− 0.01	0.98	− 1.96, 1.94	109.83	− 0.01	0.99	− 0.002
	6-month intervention	ET ET-Adapted	− 0.78	1.02	− 2.79, 1.24	92.48	− 0.76	0.45	− 0.12
	12-month intervention	ET ET-Adapted	− 1.00	1.05	− 3.10, 1.11	65.89	− 0.95	0.35	− 0.15
PSOC satisfaction	Post intervention	ET ET-Adapted	1.85	1.18	− 0.50, 4.20	108.02	1.56	0.12	0.27
	6-month intervention	ET ET-Adapted	2.01	1.24	− 0.60, 4.48	90.98	1.62	0.11	0.30
	12-month intervention	ET ET-Adapted	4.09	1.37	1.36, 6.82	71.48	2.98	0.004*	0.61
PSOC efficacy	Post intervention	ET ET-Adapted	− 2.01	0.92	− 3.83, − 0.18	107.74	− 2.18	0.03*	− 0.38
	6-month intervention	ET ET-Adapted	− 0.32	1.06	− 2.42, 1.78	83.35	− 0.31	0.76	− 0.06
	12-month intervention	ET ET-Adapted	1.11	1.60	− 2.09, 4.31	60.36	0.70	0.49	0.21
PSI-SF total	Post intervention	ET ET-Adapted	− 1.87	2.51	− 6.86, 3.12	87.19	− 0.74	0.46	− 0.11
	6-month intervention	ET ET-Adapted	− 3.93	3.82	11.56, 3.71	66.35	− 1.03	0.31	− 0.23
	12-month intervention	ET ET-Adapted	− 3.18	3.42	− 10.03, 3.68	54.60	− 0.93	0.36	− 0.18

All analyses used raw scores

*d* = effect size calculation comparing ET to ET-Adapted; a positive value indicates that ET reduced the score more than ET-Adapted and a negative value indicates that ET-Adapted reduced the score more than ET

*ET* Exploring Together; *ET-Adapted* Exploring Together-Adapted; *APQ* Alabama Parenting Questionnaire; *PSI-SF* Parent Stress Index-Short Form; *PSOC* Parenting Sense of Competence

^a^All contrasts were between the ET and ET-Adapted groups

*p < 0.05. All scores adjusted for school, child gender, child age, IQ composite score (K-BIT-2), parent gender, parent age, and family income

### Parent Satisfaction with Program

Scores on the parent satisfaction questionnaire indicated that parents were satisfied to very satisfied with all components of both the ET (*M* = 3.98 to 4.76) and ET-Adapted (*M* = 4.14 to 4.79) program on a 5-point scale. An independent-samples *t*-test indicated that parents who participated in the ET-Adapted program expressed significantly higher satisfaction with the children’s group (ET: *M* = 4.34, *SD* = 0.71; ET-Adapted: *M* = 4.61, *SD* = 0.57; *t* (100) = 2.14, *p* = 0.04). Otherwise, there were no statistically significant differences between treatment groups on mean satisfaction scores reported by parents (all *p’*s > 0.05).

Pearson's correlation coefficient (*r*) tests were conducted to assess whether there was an association between change in parenting behavior and parent satisfaction with the program. There was no significant correlation between these variables at any time point.

## Discussion

This study examined the effectiveness of two versions of the Exploring Together program on parenting behavior, parenting stress and sense of competence, with (ET) and without (ET-Adapted) the parent–child interactive component. Study results provided evidence of reduction in negative parenting behavior across both treatment groups (ET, ET-Adapted) at post intervention, maintained at the 6- and 12-month follow-ups. The significant reduction in negative parenting behavior found in this study is consistent with a review of group-based parent training programs [47]. There was no evidence to suggest that the inclusion of the parent–child interactive groups in the ET program resulted in superior change in negative parenting behavior compared to the ET-Adapted program. However, this study’s demonstration of a 12-month maintenance of treatment effect on negative parenting behavior is an important outcome.

Results indicated no significant improvement in positive parenting behavior across both treatment groups (ET, ET-Adapted). This finding is consistent with other studies of group-based parenting programs that demonstrated significant reduction in negative parenting behavior but no significant improvement in parent self-report of positive parenting behavior [98, 99]. However, this finding is in contrast with a review [47] and some studies of group-based parenting programs [100–102]. This contradictory finding could be due to parents’ high self-report of positive parenting behavior prior to treatment. Study findings indicated that children who were invited to participate in treatment were less socio-economically disadvantaged compared to children who did not receive treatment. As compared to higher socio-economic family environments, parenting within low socio-economic family environments has been observed to demonstrate lower levels of positive parenting behaviors [103]. Therefore, it is possible that the lack of significant improvement for positive parenting behavior may have occurred because parents excluded from the study would perhaps report lower levels of positive parenting. Rather than relying solely on parent self-report of parenting behaviors, future studies could also use independent observational measures to explore change in behaviors as an outcome of parent training.

Total parenting stress on the PSI-SF did not decrease significantly across both treatment groups (ET, ET-Adapted). This result conflicts with findings from a review of group-based parent training programs [49]. However, in this study the baseline mean PSI-SF total scores for participants in both treatment groups (ET, ET-Adapted) were below the cut-off for the range considered to be clinically significant [94]. Children who were invited to participate in treatment were less socio-economically disadvantaged than children who met SDQ eligibility criteria but were not invited to receive treatment. As previous research has shown that families with a higher socio-economic status experience fewer stressors compared to those with a lower socio-economic status [104], results may have been influenced by the demographic composition of the sample. Previous research has also shown social/partner support to be a potential mitigator of parenting stress [33, 34]. As such, participants in this sample may have experienced higher levels of social/partner support in their parenting role compared to other parents, which may partially explain lower levels of parenting stress reported by parents at baseline.

Significant improvement was found for parenting self-efficacy across both treatment groups (ET, ET-Adapted), maintained up to six-months after program completion. This benefit of both versions of the Exploring Together program is in keeping with a previous review that found evidence of a significant benefit of group-based intervention programs on parental self-efficacy [50]. Given that parental self-efficacy has been demonstrated to directly affect the quality of parenting behavior [43], findings support the benefit of strengthening self-efficacy in parenting programs that provide active skills training for parents and teach parents how to improve their relationships with their children. There was a significant difference between groups on parental self-efficacy at post intervention. However, caution is warranted in interpreting this finding due to a lack of significant difference between groups at the 6- and 12-month follow-ups and sample attrition at 12-month follow up.

The PSOC parenting satisfaction scale examines parent’s affective dimensions of anxiety, frustration and motivation in relation to parenting their child. This study found no significant improvement in parenting satisfaction across both treatment groups (ET, ET-Adapted). This finding is in keeping with another group parent training program for children with attention deficit/hyperactivity disorder (ADHD) [105]. However, this finding is in contrast with other evaluations of parent training programs that reported significant improvements in parenting satisfaction post treatment [106, 107]. It is likely that parenting satisfaction did not show improvement as scores at baseline approximated a community sample [37]. There was significantly greater improvement in parenting satisfaction at the 12-month follow-up for parents in the ET-Adapted program compared to the ET program. Further investigation into the longer-term impact of the Exploring Together program for parenting satisfaction as parents to continue to navigate the challenges of parenting may be warranted [108].

In summary, this study found significant improvement in negative parenting behavior and parenting self-efficacy across both treatment groups (ET, ET-Adapted). However, contrary to study hypotheses, the ET program (inclusive of parent–child interactive groups) did not result in greater change in negative parenting behavior and parenting self-efficacy compared to participants who completed the ET-Adapted program (without the parent–child interactive groups). Several possible explanations for these findings are provided. First, studies included in prior reviews [56, 58] that incorporated a parent–child interactive component [109, 110] had a higher treatment dosage (i.e., greater number of hours spent in the parent–child interactive component of treatment) compared to the current study. Second, compared to prior studies of group-based parent training programs inclusive of a parent–child interactive component [62, 111], the ET program implemented in this study did not require participants to master parent–child relationship skills (i.e., child-centred skills, decreased leading and directive parent behavior, effective commands) during the parent–child interactive component. Third, the results of the current study relied exclusively on parent self-report measures. This may have limited the capacity for the ET program to demonstrate added benefit over and above the ET-Adapted program.

It is possible that group delivery of the parent–child interactive component of the program may have impacted on learning. With up to eight parent–child dyads in the group, parents and children may have experienced increased social pressure and/or anxiety as they learned and practiced new relationship skills in front of other parent–child dyads [112, 113]. Further, some studies involving delivery of a parent–child interactive component in a group environment have included fewer parent–child dyads [111–113]. Therefore, it is possible that because of the larger number of parent–child dyads included in the parent–child interactive component in this study, there was not enough time for each parent to receive direct coaching and feedback. Also, as this program was delivered in schools, children may have participated in the parent–child interactive component with their peers, which may have increased their self-consciousness and impacted on learning [114, 115]. Future studies may therefore consider delivering the parent–child interactive component of the program on an individual basis.

This study demonstrated that the two versions of Exploring Together were equally effective. One way to interpret this result is to conclude that the in vivo parent–child interactive groups are not a necessary additional component of the program. Further, factors related to treatment dosage and mastery of parent–child relationship skills may have limited the capacity for the ET program to demonstrate added benefit in terms of parenting outcome variables relative to the ET-Adapted program. It is also possible that there were benefits of either version of the program that were not captured by the outcome measures used. For instance, the parent–child interactive component of the program may have benefits such as increased warmth and security between parents and children due to increased use of positive attention strategies. Although the two versions of Exploring Together were found to be equally effective in this study, notwithstanding study limitations, future studies may benefit from including additional independent observational measures of the parent–child relationship and interaction rather than reliance on parent self-report [116].

The current study had a number of strengths and closely adhered to guidelines recommended for cluster-randomized trials [73]. A key strength of the study was the random assignment of schools, a particularly salient feature given that previous studies of the Exploring Together program did not involve random assignment [53, 60]. In regard to implementation effectiveness, a high level of treatment satisfaction was reported by parents for all aspects of both versions of the Exploring Together program, an important indicator of parent acceptability of the program. A high rate of average attendance by parents at the weekly treatment sessions across both treatments (ET, ET-Adapted) suggests good parent engagement with the program.

Current study findings must be considered in the context of some limitations. Attrition at the longer-term follow-ups was moderate to high, thus reducing sample size and statistical power. Conclusions drawn from the study should therefore be interpreted with caution. Study results relied exclusively on parent self-report, which does not exclude the possibility of changes occurring due to parent’s perception of the situation. Further, parents were not blinded to school treatment allocation, which could introduce bias due to expectancy effects. Rather than relying solely on parent self-report of parenting behaviors, future trials of the Exploring Together program would benefit from including independent observational measures to explore change in behaviors as an outcome of parent training [100].

A potential methodological limitation of the study concerns treatment fidelity. Some treatment group leaders did not complete the 2-day training workshop but instead were trained via a ‘train the trainer’ co-facilitation method. This may have impacted on fidelity of delivery of the program. A number of factors impact the capacity to generalize the findings of this study. Families who had a recent or current significant change in family circumstances or significant parental mental health problems were excluded. It is acknowledged that these parents may well have benefited from the intervention. Findings suggest that children who were invited to participate in treatment were less socio-economically disadvantaged compared to children who did not receive treatment. Therefore, findings may not generalize to socio-economically disadvantaged families.

The research presented in this study has important implications for future research involving group-based parent training programs in primary school settings [117]. Given disparities in the use of community-based mental health services for children and families [118], future trials could encourage participation from parents across the socioeconomic spectrum by offering flexible scheduling options, childcare, and transportation assistance, and delivery of the program outside school hours [119]. This would likely involve complex and coordinated efforts between mental health services, school leadership staff, and other existing resources at the school to achieve such impact.

## Summary

The Exploring Together program is a group-based parent training program comprised of separate parent, child, and teacher components, and a combined parent–child interactive component. The current study aimed to compare the effectiveness of two versions of the Exploring Together program for improving parenting behavior, parenting stress and parenting sense of competence, associated with (Exploring Together; ET) and without (Exploring Together-Adapted; ET-Adapted) the parent–child interactive component. One hundred and thirty-six parents and their children (aged 5–10 years) with externalizing and/or internalizing problems participated in the trial, recruited from primary schools. Parents were administered self-report measures of parenting behavior, parenting stress and sense of competence, assessed at post intervention, 6-month and 12-month follow-up. This study provided evidence of significant reductions in negative parenting behaviors but no significant improvement in positive parenting behavior for participants of both versions of the Exploring Together program. Parenting self-efficacy improved significantly across both treatment groups (ET, ET-Adapted), however, there was no significant change in parenting satisfaction or parenting stress. Consistent with child outcomes in terms of externalizing and internalizing problems, the inclusion of the parent–child interactive component (ET program) did not result in superior treatment outcomes relative to the version (ET-Adapted program) that omitted this component [69]. Parent engagement and treatment satisfaction with both versions of the program was high. It is possible that the lack of difference between treatment groups was due to insufficient time for each parent to receive direct coaching and feedback or achieve superior mastery of parenting skills during the parent–child interactive groups. Future trials of the Exploring Together program would benefit from the inclusion of parents across the socioeconomic spectrum and those with mental health difficulties which may require a coordinated strategy involving mental health and school personnel. In addition, trial methodology would be strengthened by including additional assessment methods, such as independent observations of parenting behaviors. Further investigation regarding treatment dosage and mastery of parent–child relationship skills associated with the program is warranted.
